# Quantitation of cancer treatment response by 2-[^18^F]FDG PET/CT: multi-center assessment of measurement variability using AUTO-PERCIST™

**DOI:** 10.1186/s13550-021-00754-1

**Published:** 2021-02-12

**Authors:** Joo Hyun O, Su Jin Lim, Hao Wang, Jeffrey P. Leal, Hui-Kuo G. Shu, Richard L. Wahl, QIN PET Readers

**Affiliations:** 1 Department of Radiology, Seoul St. Mary’s Hospital, College of Medicine, The Catholic University of Korea, Seoul, Korea; 2 Division of Biostatistics and Bioinformatics, Department of Oncology, Johns Hopkins University School of Medicine, Baltimore, MD USA; 3 Division of Nuclear Medicine, The Russell H. Morgan Department of Radiology and Radiological Science, Johns Hopkins University School of Medicine, Baltimore, MD USA; 4 Department of Radiation Oncology, The Emory Clinic, Atlanta, GA USA; 5 Mallinckrodt Institute of Radiology, Washington University, St. Louis, MO USA; 6 Washington University School of Medicine, Mallinckrodt Institute of Radiology, 510 South Kingshighway Blvd, Campus Box 8131, St. Louis, MO 63110 USA

**Keywords:** [^18^F]FDG PET/CT, Response assessment, Quantification

## Abstract

**Background:**

The aim of this study was to assess the reader variability in quantitatively assessing pre- and post-treatment 2-deoxy-2-[^18^F]fluoro-d-glucose positron emission tomography/computed tomography ([^18^F]FDG PET/CT) scans in a defined set of images of cancer patients using the same semi-automated analytical software (Auto-PERCIST™), which identifies tumor peak standard uptake value corrected for lean body mass (SUL_peak_) to determine [^18^F]FDG PET quantitative parameters.

**Methods:**

Paired pre- and post-treatment [^18^F]FDG PET/CT images from 30 oncologic patients and Auto-PERCIST™ semi-automated software were distributed to 13 readers across US and international sites. One reader was aware of the relevant medical history of the patients (read_reference_), whereas the 12 other readers were blinded to history but had access to the correlative images. Auto-PERCIST™ was set up to first automatically identify the liver and compute the threshold for tumor measurability (1.5 × liver mean) + (2 × liver standard deviation [SD]) and then detect all sites with SUL_peak_ greater than the threshold. Next, the readers selected sites they believed to represent tumor lesions. The main performance metric assessed was the percent change in the SUL_peak_ (%ΔSUL_peak_) of the hottest tumor identified on the baseline and follow-up images.

**Results:**

The intra-class correlation coefficient (ICC) for the %ΔSUL_peak_ of the hottest tumor was 0.87 (95%CI: [0.78, 0.92]) when all reads were included (*n* = 297). Including only the measurements that selected the same target tumor as the read_reference_ (*n* = 224), the ICC for %ΔSUL_peak_ was 1.00 (95%CI: [1.00, 1.00]). The Krippendorff alpha coefficient for response (complete or partial metabolic response, versus stable or progressive metabolic disease on PET Response Criteria in Solid Tumors 1.0) was 0.91 for all reads (*n* = 380) and 1.00 including for reads with the same target tumor selection (*n* = 270).

**Conclusion:**

Quantitative tumor [^18^F]FDG SUL_peak_ changes measured across multiple global sites and readers utilizing Auto-PERCIST™ show very high correlation. Harmonization of methods to single software, Auto-PERCIST™, resulted in virtually identical extraction of quantitative tumor response data from [^18^F]FDG PET images when the readers select the same target tumor.

## Introduction

2-Deoxy-2-[^18^F]fluoro-d-glucose positron emission tomography/computed tomography ([^18^F]FDG PET/CT) is increasingly applied in monitoring treatment response in patients with cancer. While PET is intrinsically a quantitative imaging technique, many PET assessments of cancer response are qualitative, as, for example, in lymphoma where quantitative PET data are converted into a five-point qualitative scale which is practical and highly useful [1, 2]. Quantitative PET assessments of response have been deployed in many research imaging studies, especially in examining early treatment response-related changes in metabolism including breast cancer where these changes can predict much later pathological outcomes [3, 4]. The PET Response Criteria in Solid Tumors 1.0 (PERCIST 1.0) were proposed in 2009 as a method to standardize the assessment of tumor response on [^18^F]FDG PET and emphasized use of the peak standard uptake value corrected for lean body mass (SUL_peak_) in contrast to the maximum standardized uptake value (SUV_max_) [5, 6]. The SUV_max_ is reasonably easy to determine with many forms of software, while the SUL_peak_ is more challenging to measure [7].

Thus, despite its attractiveness, quantitative PET utilizing PERCIST is not routinely performed for assessing response to therapy in patients with cancer in the clinic or many clinical trial settings, contrary to the routinely utilized Response Evaluation Criteria in Solid Tumors for assessment of anatomical imaging. One way to expand the use of quantitative [^18^F]FDG PET/CT in clinical trials and clinical practice is to reduce reader variability of SUV measurements and make the measurements rapid and automated. In a previous multi-center, multi-reader study we conducted, multiple sites assessed the same paired pre- and post-treatment [^18^F]FDG PET/CT images in cancer patients. The intra-class correlation coefficient (ICC) of percent change in SUV_max_ was 0.89 (95% confidence interval (CI): [0.81, 0.94]) across multiple performance sites using a variety of analytical software tools. The ICC for the SUL_peak_ was lower at 0.70 (95% CI: [0.54, 0.80]). SUL_peak_ is, in principle, the more statistically sound of the PET parameters, and it is the suggested metric in PERCIST [7]. However, if there is considerable variability among sites in how SUL_peak_ is generated and measured, then the PERCIST metric potentially may introduce variability into assessments of treatment response, as opposed to reducing variability [8].

The aim of the present study was to determine whether the utilization of Auto-PERCIST™, a semi-automated software system for the quantitative assessment [^18^F]FDG PET images, could lower the reader variability in quantitatively assessing pre- and post-treatment [^18^F]FDG PET/CT studies for response in a multi-center, multi-reader, multi-national study assessing identical images.

## Materials and methods

Pre- and post-treatment [^18^F]FDG PET/CT images of 30 oncologic patients selected from a group of tumor types having representative patterns of FDG avidity contained a mix of single and multiple tumors on the pretreatment scan (1 tumor, *n* = 6; > 1 but < 10 tumors, *n* = 19; ≥ 10 tumors, *n* = 5), and a mix of the four major response categories using PERCIST (complete metabolic response, *n* = 6; partial metabolic response, *n* = 11; stable metabolic disease, *n* = 4; and progressive metabolic disease, *n* = 9).

Sites both with National Cancer Institute Quantitative Imaging Network affiliation and without which did not participate in the previous study with the same data set were recruited by e-mail and conference calls. The dataset was the based on a previous study of reader variability [9].

Thirty anonymized cases of pre- and post-treatment [^18^F]FDG PET/CT studies (total 60 studies) were distributed along with directions for installing and utilizing the Auto-PERCIST™ software. Approval from the Johns Hopkins Institutional Review Board was obtained, and the need for patient informed consent was waived for this study of anonymized image data.

### Measurement

Individual measurements from coupled pre- and post-treatment [^18^F]FDG PET/CT images from one patient were counted as a *read*. The coupled pre- and post-treatment measurements for all 30 cases from a single reader were counted as a *set of reads*. One reader from the central site (reader 1) had full knowledge of the primary tumors, treatment histories and subsequent follow-up results, but all other readers had no knowledge of the patients’ medical histories as the reader is often intentionally blinded in the setting of multi-center trials. For statistical purpose, the measurements by reader 1 were considered as the reference standard for comparison (read_reference_).

**Fig. 1:**
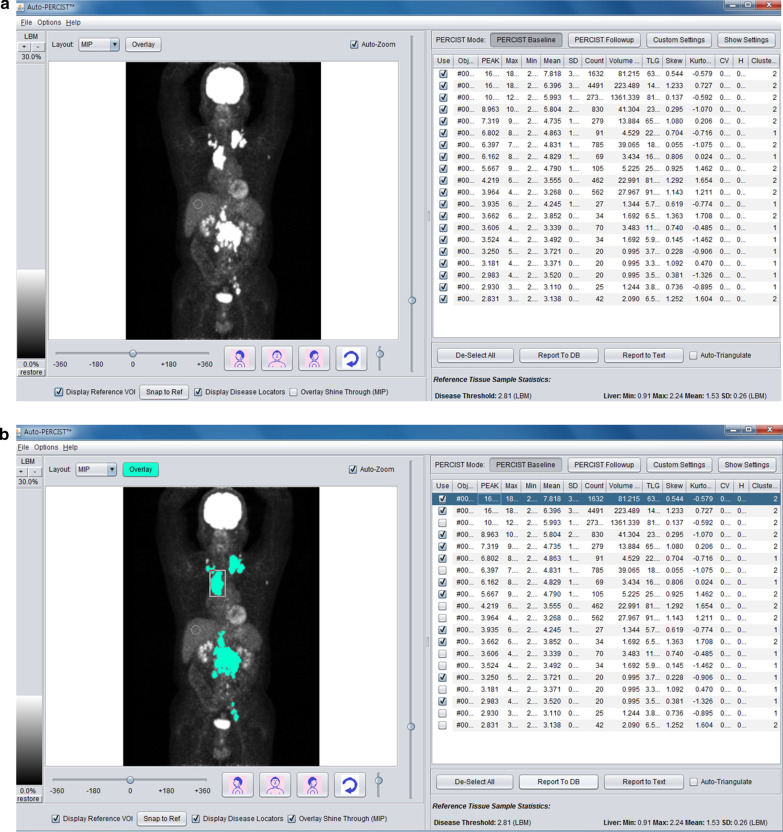
Screen captures from Auto-PERCIST™. **a** The software detected all sites with SUL_peak_ higher than the computed or manually set threshold. **b** The reader than selected the true tumor lesions (shaded in green), excluding physiologic [^18^F]FDG activity

Each reader determined which tumor to measure. The Auto-PERCIST™ loads the PET images and automatically obtains liver measurements from a 3-cm-diameter sphere in the right side of the liver to compute the threshold for lesion detection. The default setting is 1.5 × liver mean + 2 standard deviations (SD) at baseline to ensure the decline in [^18^F]FDG uptake is less likely due to chance and to minimize overestimation of response or progression. For follow-up images, the default setting is lower at 1.0 × liver mean + 2SD, to allow detection of lesions with lower SULpeak. If a lesion was perceptible visually but not detected using the default threshold settings, the reader had the choice to manually lower the threshold for detection. The Auto-PERCIST™ would detect all sites with SUL_peak_ higher than the threshold (Fig. 1). It was up to the readers to determine whether the detected sites were true tumor lesions or not. The reader could also separate a detected focus of [^18^F]FDG uptake into separate smaller lesions when needed—to exclude adjacent physiologic [^18^F]FDG uptake or break down a large conglomeration of tumors into smaller separate lesions. The reader could also add smaller [^18^F]FDG uptake lesions to make them a single lesion if the reader decided the separate [^18^F]FDG uptakes were parts of a larger single lesion. The readers were instructed to select up to 5 of the hottest tumors for cases with multiple lesions. The readers could view the PET/CT images on any reading software they preferred, but the measurements came only from the Auto-PERCIST™. The measurements from Auto-PERCIST™ included SUL_peak_, maximum and mean SUL, number of counts, geometric mean, exposure, kurtosis, skewness and metabolic volume. After the readers selected and quantified the lesions, the measurements were saved as text files and sent for central compilation and analysis to the Image Response Assessment Core at Johns Hopkins University.

### Statistical analysis

The primary study metric was the percentage change in SUL_peak_ (%ΔSUL_peak_) from baseline to follow-up. Percentage change was defined as [(follow-up measurement − baseline measurement)/(baseline measurement)] × 100. For assessment of up to five lesions, the percentage change was computed from the sum of the lesions. Treating both case and site as random effects, a linear random-effects model was fit via the restricted maximum likelihood estimation method, which estimated variance components of the random effects in the model. As a measure of inter-rater agreement, the intra-class correlation coefficient (ICC) was computed using the variance components of the random effects. The ICC was computed as [inter-subject variance/(inter-subject variance + intra-subject variance + residual variance)]. The bias-corrected and accelerated bootstrap method was implemented with 1,000 bootstrap replicates to construct the 95% confidence interval of the computed ICC. The sampling unit was a *read*.

To assess agreement between the reference reader (read_reference_) and another reader, the ICC was computed for each pair of the reference reader and 12 other readers. The mean of these ICCs and its range (minimum, maximum) were reported.

Krippendorff alpha reliability coefficient was computed as a measure of agreement between multiple readers for response outcome, which was classified into four ordered major response categories using PERCIST 1.0 as: complete metabolic response (CMR), partial metabolic response (PMR), stable metabolic disease (SMD) and progressive metabolic disease (PMD). The measurements were classified: PMD for SUL_peak_ increase ≥ 30% (and 0.8 units) or new lesions; SMD for SUL_peak_ increase or decrease < 30% (or 0.8 units); PMR for SUL_peak_ decrease ≥ 30% (and 0.8 units); and CMR for no perceptible tumor lesion. Additionally, Krippendorff coefficient was computed with the response categories being dichotomized into two levels: clinical benefit (CMR/PMR/SMD) and no benefit (PMD) or response (CMR/PMR) and no response (SMD/PMD). Krippendorff suggests 0.8 as a threshold for satisfactory reliability, but if tentative conclusions are acceptable, 0.667 is the lowest conceivable threshold [10].

## Results

### All *reads*

*Reads* were received from 13 different sites from January to September of 2018. A single reader (nuclear medicine physician/radiologist/radiological scientist) at each site measured all 30 cases. Measurements were treated as missing when a reader did not submit data.

**Fig. 2:**
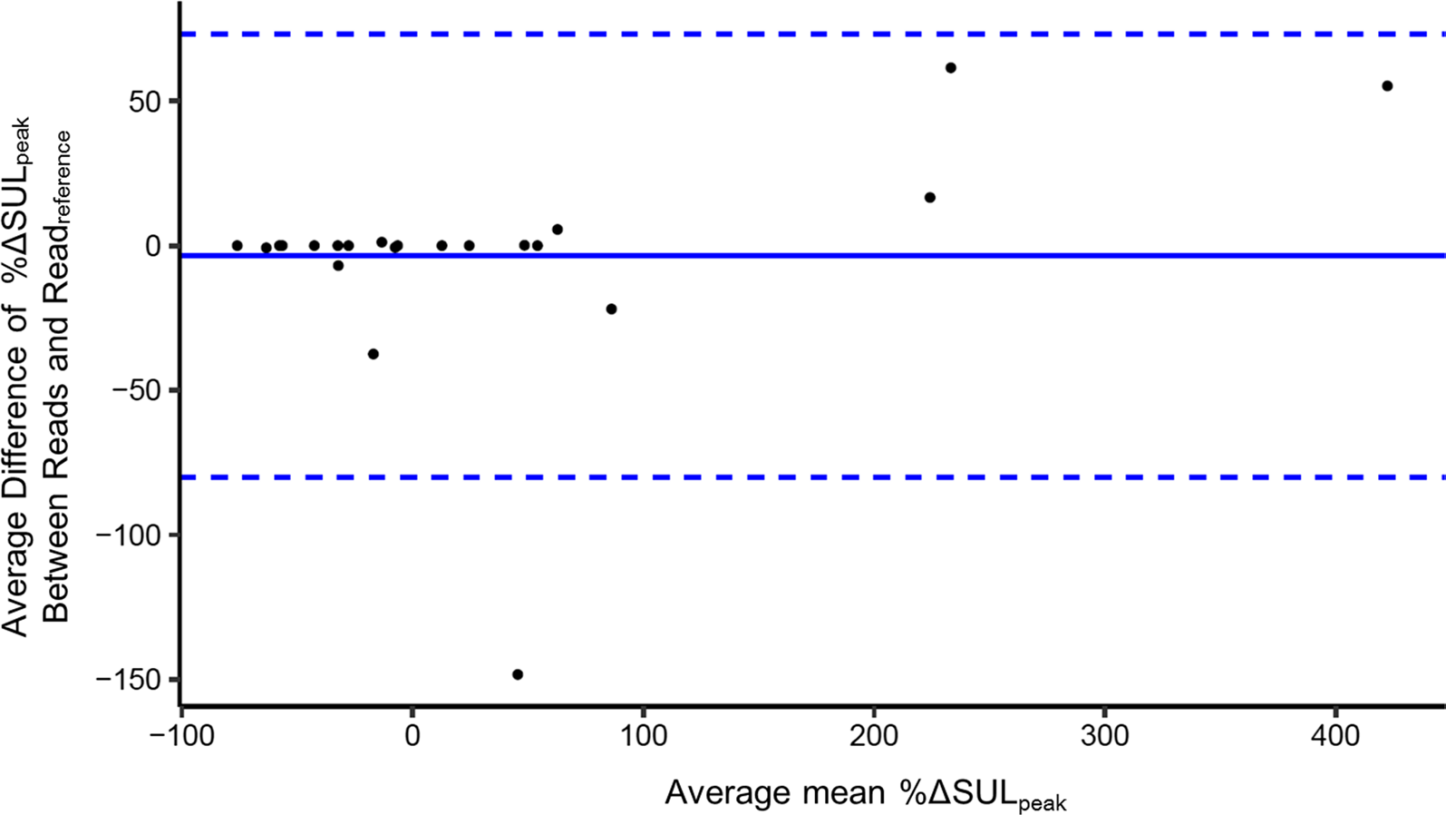
Bland–Altman plot of the percentage change of tumor [^18^F]FDG uptake from baseline to follow-up. The plot is for the percentage changes of SUL_peak_ for all reads. Each dot represents a case (30 cases in total). The x-axis represents the average mean percentage change measurement by all readers. The y-axis represents the average *difference* between the 12 readers and the reference reader (read_reference_). The solid line represents the average bias, and the dashed lines represent the corresponding bias ± 2 standard deviations (SD)

Among a total of 390 possible *reads* by 13 readers, 347 baseline *reads* and 329 follow-up *reads* were reported, of which 297 *reads* were complete baseline and follow-up pairs. Such *reads* were used to compute the ICC with all readers and agreement with read_reference_ for the baseline, follow-up and percentage change in SUL_peak_, respectively. The ICC for %ΔSUL_peak_ was 0.87 (95% CI: [0.78, 0.92]), and agreement with read_reference_ was 0.88 (range: [0.61, 1.00]). The ICC and agreement with read_reference_ of other metrics are given in Table 1. The overall within-subject coefficient of variance (COV; overall SD/average of the case means) for %ΔSULpeak change was computed as 2.293. The Bland–Altman plot of the %ΔSUL_peak_ is shown in Fig. 2.

**Table 1:** ICC and agreement for single or up to five SUL_peak_ selections

	ICC (95% CI)			Agreement with read_reference_ ICC (Min, Max)		
	Baseline	Follow-up	Percentage change	Baseline	Follow-up	Percentage change
1 SUL_peak_ (All reads)	0.90 (0.82, 0.94)	0.75 (0.64, 0.82)	0.87 (0.78, 0.92)	0.95 (0.72, 1.00)	0.78 (0.37, 1.00)	0.88 (0.61, 1.00)
1 SUL_peak_ (Reads with same tumor selected)	1.00 (0.49, 1.00)	1.00 (1.00, 1.00)	1.00 (1.00, 1.00)	0.95 (0.43, 1.00)	1.00 (1.00, 1.00)	1.00 (1.00, 1.00)
Sum of up to 5 SUL_peak_ (All reads)	0.93 (0.85, 0.96)	0.85 (0.67, 0.92)	0.77 (0.61, 0.85)	0.95 (0.84, 0.99)	0.89 (0.46, 0.99)	0.80 (0.39, 0.95)
Sum of up to 5 SUL_peak_ (Reads with same tumors selected)	0.98 (0.94, 0.98)	0.98 (0.94, 0.98)	0.96 (0.92, 0.98)	0.97 (0.95, 0.99)	0.98 (0.97, 1.00)	0.93 (0.87, 1.00)

Agreement with read_reference_—ICC between reference reader and each of the other 12 readers

ICC, intra-class correlation coefficient; CI, confidence interval; SUL_peak_, peak standardized uptake value corrected for lean body mass

### *Reads* with same target tumor

**Fig. 3:**
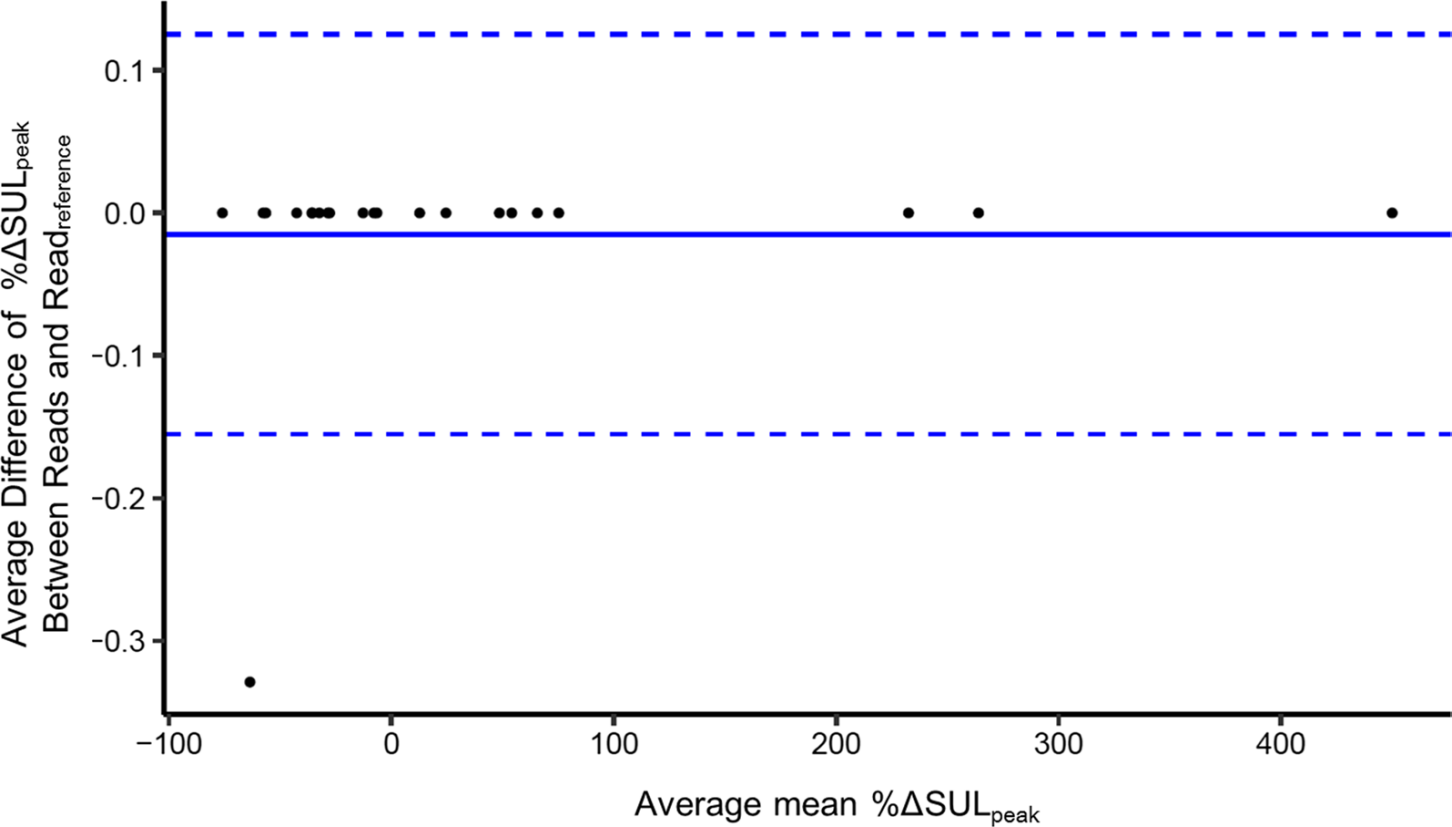
Bland–Altman plot of the percentage change of tumor [^18^F]FDG uptake from baseline to follow-up. The plot is for the percentage changes of SUL_peak_ (%ΔSUL_peak_) for *only the reads with same lesion selected* as the read_reference_. Each dot represents a case (30 cases in total). The x-axis represents the average mean %ΔSUL_peak_ measurement by all readers. The y-axis represents the average difference between the 12 readers and the reference reader (read_reference_) and the y-axis unit is one-tenth of one percent. The solid line represents the average bias, and the dashed lines represent the corresponding bias ± 2 standard deviations (SD)

Among the 360 possible *reads* from the 12 readers, the readers selected a different lesion compared to the read_reference_ in 46 *reads* at baseline, 43 *reads* at follow-up and 29 *reads* at both baseline and follow-up. The 241 *reads* agreeing on target selection with the read_reference_ were used to compute the ICCs with all readers and agreement with read_reference_. The ICC for %ΔSUL_peak_ among all readers was 1.00 (95% CI: [1.00, 1.00]), and agreement with read_reference_ was 1.00 (range: [1.00, 1.00]). The ICC and agreement with read_reference_ of other metrics are given in Table 1. The overall within-subject COV for %ΔSULpeak change was computed as 0.007. The Bland–Altman plot is shown in Fig. 3.

### Sum of up to 5 SUL_peak_

In addition to the SUL_peak_ measurement of a single lesion, the sum of SUL_peak_ measurements of up to 5 of the selected lesions was used to compute the ICC and agreement with read_reference_ for all reads and reads with the same target lesion (Table 1). Even when the same lesions were selected, the ICCs and agreement with read_reference_ were not a perfect 1.00 due to (a) differences in the manual thresholds used for lesion detection and (b) utilization of the ‘erosion option’ for breaking up [^18^F]FDG uptake volumes by the individual readers.

### Inter-rater reliability of readers on responses

Among the 390 *reads* for all reads, 380 *reads* reported response categories. Among the 271 *reads* agreeing on target selection with the read_reference_, 270 reported response categories. The Krippendorff alpha coefficient of 13 readers for binary response measure (response (CMR/PMR) versus no-response (SMD/PMD)) was 0.91 for all *reads* and 1.00 for only the *reads* with the same target lesion selection. When assessing clinical benefit (SMD/PMR/CMR representing clinical benefit versus PMD representing no benefit), the Krippendorff alpha coefficient was 0.81 for all *reads* and 1.00 for only the *reads* with the same target selection. With the four response categories treated in an ordinal scale, the Krippendorff alpha coefficient was 0.86 for all *reads* and 1.00 for only the *reads* with the same target selection (Table 2).

**Table 2:** Inter-rater reliability of readers on response assessment

	Krippendorff alpha coefficient of 13 readers on response	
	All reads (*n* = 380^a^)	Reads with same target (*n* = 270^a^)
Response vs. no response (CMR/PMR vs. SMD/PMD)	0.91	1.00
Clinical benefit vs. no benefit (CMR/PMR/SMD vs. PMD)	0.81	1.00
Response categories (ordinal scale) CMR vs. PMR vs. SMD vs. PMD	0.86	1.00

^a^*Reads* with missing response were excluded (10 for all reads and 1 for reads with same target)

## Discussion

Variability in measurements across readers and sites is an often cited hurdle to broader utilization of quantitative [^18^F]FDG PET/CT for response assessment of cancer treatment [11]. Test–retest studies have demonstrated high repeatability of [^18^F]FDG and other radiopharmaceutical PET parameters [12–15]. The variance of SUVs could be greater in clinical practice compared to ideal study setting [16]. In the clinical setting, measurement of SUV_max_ was demonstrated to have high agreement in our previous paper, while the statistically more robust SUL_peak_ showed suboptimal agreement [9]. We wanted to know whether using uniform software could eliminate the variability associated with the computation differences for SUL_peak_ across multiple vendors and software.

The localization of the liver, SUL measurements from the liver, computation of a threshold for lesion detection and identification of candidate lesions were all performed automatically on Auto-PERCIST™. Following detection of all sites with SUL_peak_ higher than the set threshold, various [^18^F]FDG uptake intensity or pattern measurements and textural features for each of the detected sites were also performed automatically. When the readers chose the same single target tumor, the measurements were identical, as could be expected. For up to five hottest lesions measurements, the agreement was near perfect. However, agreement was not a perfect 1.00 even when the readers chose the same tumors because the readers had the option to break down a single volume of [^18^F]FDG uptake to separate parts, or add up two or more [^18^F]FDG uptake sites to a single volume as they determined appropriate. Some readers chose to break down a lesion detected on Auto-PERCIST™ to avoid including physiologic [^18^F]FDG uptake, or to separate a conglomeration of multiple tumors lesions. And some readers intentionally chose a detection threshold lower than the default software setting to include lesions with relatively low [^18^F]FDG uptake for assessment on the follow-up PET images. The agreement was lower for follow-up images for the all-reads assessment. The readers disagreed more often on what was tumor and what was physiologic or inflammatory response on the follow-up images.

A previous paper that showed excellent correlation between two different vendor software tools for SUL_peak_ had the tumor sites predefined by the readers to exclude interpretive error [13]. Determining which [^18^F]FDG uptake site is true tumor remained a challenge even for experienced readers. In the outlier case in Fig. 2 showing an average difference greater than 100%, some readers considered an intense [^18^F]FDG uptake in the colon on the follow-up image to be new tumor lesion, while the read_reference_ considered it physiologic in nature. Of the 360 non-reference baseline reads (including missing measurements) in this study, only 241 reads (67%) chose the same lesion and went on to make the same measurements as the read_reference_ at both baseline and follow-up. Among the 30 cases, the target lesion (hottest tumor) on the post-therapy scan was different from the target lesion noted on the pre-therapy scan in 11 cases. For example, in one case, the target lesion was in a mediastinal node on pre-therapy scan, and then, a lung lesion became the hottest tumor in the post-therapy scan. In three cases, nodes in different stations were the target lesions at different time points. Among patients with multiple bone or lung metastases, different lesions in the same organ could be observed becoming the target tumors at different time points. As seen in inter-observer agreement studies of [^18^F]FDG PET/CT performed in patients with lymphoma after therapy, even experienced readers do not always agree on what is tumor [^18^F]FDG uptake and what is physiologic [^18^F]FDG uptake [17, 18]. Rather than relying solely on the reading experience of the local site, discussions and consensus meetings and better training methods are necessary to implement [^18^F]FDG PET/CT to its full potential. It almost certainly is the case that the availability of more relevant patient history would result in better accuracy and consistency in tumor detection.

While PERCIST 1.0 is quantitative, the category of CMR is dependent on the reader’s judgment, and software quantification alone could not determine the response to be CMR. There were six cases considered to have reached CMR by the read_reference_. The 12 other readers categorized the case correctly as CMR in 44 *reads* out of 72 (12 readers × 6 cases), PMR was designated in 21 *reads*, SMD in 5 *reads* and PMD in 1 *read*, with 1 missing *read.* Thus, in addition to selection of different target tumors from the read_reference_, the reader’s decision between CMR and PMR leaves room for variability in response categorization, even if quantitation produces identical results. Detailed definition or consensus on findings compatible with the CMR category, or addition of quantitative threshold to clarify the CMR category, is necessary for use in trials and in the clinical setting. A lesion could be considered present and thus not CMR even with very low SUL_peak_, for example, in the lungs, or a lesion could be considered resolved and thus CMR even with relatively high SULpeak, for example, in tonsils. The threshold computed from liver measurements (liver SUL_mean_ + 2SD) was viewed by the readers as too high a cutoff for CMR in this study as could be inferred by how the readers manually lowered the threshold on the follow-up images.

**Fig. 4:**
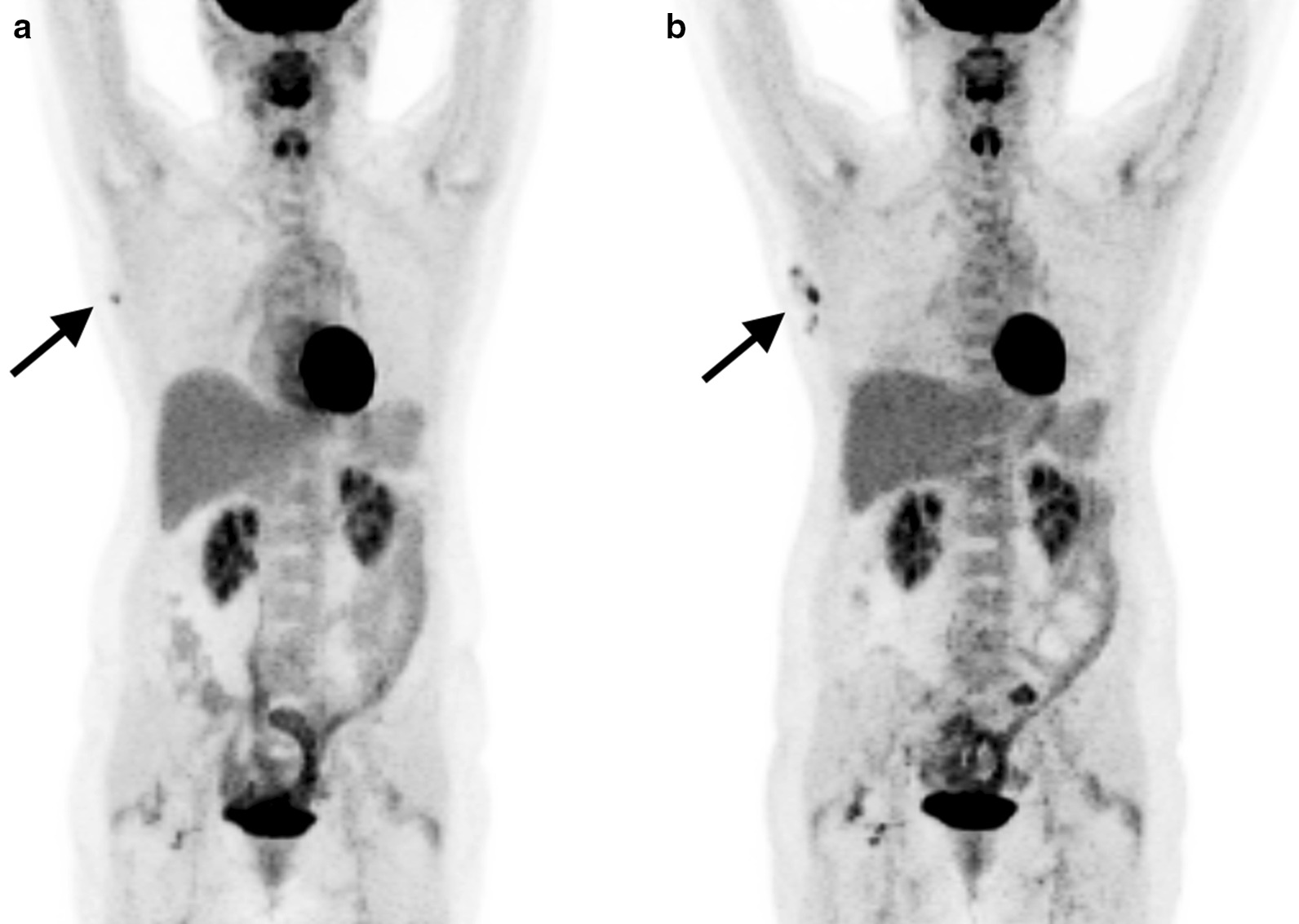
**a** PET maximum intensity projection (MIP) image of patient with right axillary node metastasis at baseline with SUL_peak_ of 1.62 and tumor volume of less than 1.00 cc. Though visually perceptible, Auto-PERCIST™ failed to detect the lesion due to small size. **b** On the follow-up MIP image, the number of metastatic nodes and the [^18^F]FDG uptake intensity are increased to SUL_peak_ of 2.84, allowing detection by Auto-PERCIST™

Revealing a potential limitation in the software, and of the PERCIST criteria, there was a small tumor with clearly perceptible [^18^F]FDG uptake visually, which was not detectable by Auto-PERCIST™ due to the volume below the PERCIST definition of SUL_peak_ sphere of 1 cubic centimeter (Fig. 4). More mundane limitation of applying PERCIST includes the need to measure the patient’s height. That many of the referring physicians and radiologist are not familiar with the SUL_peak_ parameters is another limitation to overcome. When there are multiple lesions showing intense [^18^F]FDG uptake, the lesion with the worst response may not be the target lesion, and PERCIST needs to specify how to address such poorly behaving lesions for categorizing the overall response.

Auto-PERCIST™ has the ability to automatically detect potentially new lesions for co-registered studies based on the location of the classified lesions. Auto-PERCIST™ also computed additional PET parameters representing tumor features, such as metabolic tumor volume, geometric mean, exposure, kurtosis and skewness, which have been reported as prognostic markers and diagnostic tools [19–22]. Discordance among readers was minimal for the additional PET parameters, and the cause for any variance arose when the reader manually changed the tumor boundary. Even with the addition of several PET parameters, the measurement took seconds to at the longest and a few minutes for cases with many lesions. In addition to reducing variability in measurement, the software reduced the measurement time radically. Auto-PERCIST™ may become adjunct reading software the way myocardial perfusion and metabolism studies utilize cardiac image analysis software. Auto-PERCIST is available to academic researchers who register their interest with the Johns Hopkins Technology Transfer office.

## Conclusion

Harmonization of methods to single software Auto-PERCIST™ resulted in virtually identical extraction of quantitative data including the SUL_peak_ when the readers selected the same target tumor, and should promote greater use of [^18^F]FDG PET/CT for response assessment in cancer treatment. Nonetheless, the findings show caution remains in order as lesion selection still depends on qualitative assessments of whether a lesion is tumor or physiological uptake.

## Data Availability

The datasets used in this study are available from the corresponding author on reasonable request.

## Abbreviations

[^18^F]FDG: 2-Deoxy-2-[^18^F]fluoro-d-glucose
PET/CT: Positron emission tomography/computed tomography
SULpeak: Peak standard uptake value corrected for lean body mass
%ΔSULpeak: Percent change in the SULpeak
PERCIST 1.0: PET Response Criteria in Solid Tumors 1.0
SUVmax: Maximum standardized uptake value
CI: Confidence interval
SD: Standard deviation
ICC: Intra-class correlation coefficient
CMR: Complete metabolic response
PMR: Partial metabolic response
SMD: Stable metabolic disease
PMD: Progressive metabolic disease
COV: Coefficient of variance
